# Development and validation of early warning score systems for COVID‐19 patients

**DOI:** 10.1049/htl2.12009

**Published:** 2021-05-27

**Authors:** Alexey Youssef, Samaneh Kouchaki, Farah Shamout, Jacob Armstrong, Rasheed El‐Bouri, Thomas Taylor, Drew Birrenkott, Baptiste Vasey, Andrew Soltan, Tingting Zhu, David A. Clifton, David W. Eyre

**Affiliations:** ^1^ Department of Engineering Science Institute of Biomedical Engineering University of Oxford Oxford UK; ^2^ Centre for Vision, Speech, and Signal Processing University of Surrey Guildford UK; ^3^ Engineering Division New York University Abu Dhabi Abu Dhabi United Arab Emirates; ^4^ Big Data Institute Nuffield Department of Population Health University of Oxford Oxford UK; ^5^ Stanford School of Medicine Stanford University Palo Alto USA; ^6^ Nuffield Department of Surgical Sciences University of Oxford Oxford UK; ^7^ John Radcliffe Hospital Oxford University Hospitals NHS Foundation Trust Oxford UK; ^8^ Division of Cardiovascular Medicine Radcliffe Department of Medicine John Radcliffe Hospital University of Oxford Oxford UK; ^9^ Oxford‐Suzhou Centre for Advanced Research Suzhou China

## Abstract

COVID‐19 is a major, urgent, and ongoing threat to global health. Globally more than 24 million have been infected and the disease has claimed more than a million lives as of November 2020. Predicting which patients will need respiratory support is important to guiding individual patient treatment and also to ensuring sufficient resources are available. The ability of six common Early Warning Scores (EWS) to identify respiratory deterioration defined as the need for advanced respiratory support (high‐flow nasal oxygen, continuous positive airways pressure, non‐invasive ventilation, intubation) within a prediction window of 24 h is evaluated. It is shown that these scores perform sub‐optimally at this specific task. Therefore, an alternative EWS based on the Gradient Boosting Trees (GBT) algorithm is developed that is able to predict deterioration within the next 24 h with high AUROC 94% and an accuracy, sensitivity, and specificity of 70%, 96%, 70%, respectively. The GBT model outperformed the best EWS (LDTEWS:NEWS), increasing the AUROC by 14%. Our GBT model makes the prediction based on the current and baseline measures of routinely available vital signs and blood tests.

## 1 INTRODUCTION

COVID‐19 is a major, urgent, and ongoing threat to global health. The disease has infected millions across the globe causing a surge in demand on healthcare services. This has created a significant strain on hospital resources globally, especially on intensive care units (ICUs) and respiratory support equipment such as invasive and non‐invasive ventilators (NIVs). In such conditions, a tool to predict deterioration of patients is valuable to best allocate hospital resources and to ensure that patients are placed in the correct environment to meet their needs, for example, transferred to ICU before substantial deterioration. Given the limited resources there is a significant need to prioritise the right patients so the resource is available for those who need it.

Deterioration prediction tools have traditionally existed in the form of Early Warning Score (EWS) systems or Physiological Track and Trigger Systems, which track physiological variables and alert for assistance when the variables surpass a predefined threshold [1]. EWS systems use an aggregate weighted scoring of vital signs and other variables [2]. Early EWS were not intended to predict adverse events, but rather to alert for deterioration that may precede adverse events. Currently, different scores have been developed, adjusted and validated to predict the adverse events themselves as a proxy for deterioration [1].

Current EWS systems are based on routinely measured physiological variables and different laboratory markers [2, 3, 4, 5, 6]. Common examples include the NEWS [6], CEWS [2], MCEWS [3], AEWS [7], LDTEWS [8], and LDTEWS:NEWS [4] (which combines both NEWS and LDTEWS), among others. The scores evaluate physiological parameters (NEWS, CEWS, MCEWS and AEWS), laboratory parameters (LDTEWS) and a combination of physiological and laboratory parameters (LDTEWS:NEWS) (Table 1) [2, 3, 4, 6, 7, 8]. The scores have been validated for different outcomes, including ICU admission, mortality, and cardiac arrest, usually within 24 h from the time of measurement (Table 1). It is unclear how EWS systems would perform in COVID‐19 patients, since these scores have been developed and validated to discriminate deterioration in a pre‐pandemic general inpatient cohort. By contrast, deterioration in the COVID‐19 inpatient cohort more commonly manifests through hypoxic respiratory failure [9]. Therefore, the EWS tools built may be less effective at predicting deterioration in COVID‐19 patients. For example, NEWS considers a binary variable for oxygen support (room air vs. oxygen support) while the rest of the variables are either continuous or categorical [6]. NEWS is, thus, not equipped to capture the variability in oxygen support levels which are a strong proxy for COVID‐19 severity. Despite the limitations, these tools are in routine use in many hospital managing patients with COVID‐19.

**TABLE 1:** Various Early Warning Scores evaluated in our study

Early Warning Score	Summary	Input data	Predicted outcome
National early warning score (NEWS) [6]	The national EWS in the UK and one of the most widely used EWS	HR, RR, supplemental O${}_{2}$, SpO${}_{2}$, SBP, temperature, level of consciousness (AVPU)	Early cardiac arrest, unanticipated ICU admission, and in‐hospital mortality
Centile‐based early warning score (CEWS) [2]	A centile‐based early warning score using continuously acquired bedside vital‐sign data	HR, RR, SpO${}_{2}$, SBP, temperature, level of consciousness (AVPU)	Cardiac arrest, unanticipated ICU admission, and in‐hospital mortality
Manual centile‐based early warning scores (MCEWS) [3]	A centile‐based early warning score using manually‐recorded data	HR, RR, supplemental O${}_{2}$, SpO${}_{2}$, SBP, temperature, level of consciousness (AVPU)	Cardiac arrest, unanticipated ICU admission, and in‐hospital mortality
Age‐based early warning score (AEWS) [7]	Age specific early warning score based on the NEWS score	HR, RR, supplemental O${}_{2}$, SpO${}_{2}$, SBP, temperature, level of consciousness (AVPU)	Cardiac arrest, unanticipated ICU admission, and in‐hospital mortality
Laboratory decision tree early warning score (LDTEWS) [8]	An early warning score (EWS) based on routinely collected laboratory tests	HGB, Alb, Na+, K+, Cr, Ur, WBC	In‐hospital mortality
LDTEWS:NEWS [4]	An EWS developed by combining (NEWS and LDTEWS) to discriminate unanticipated ICU admission	NEWS and LDTEWS input data	In‐hospital mortality and unplanned ICU admission

Some of the existing EWS systems have been validated on COVID‐19 patients [10, 11, 12, 13, 14]. Meylan et al. and Carr et al. have adjusted NEWS2 to adapt it for COVID‐19 patients [15, 16]. Carr and colleagues validated the ability of the NEWS2 score to identify severe COVID‐19 infections (defined as ICU admission or in‐hospital mortality). The study reported an initial performance of 0.628 area under the receiving operator characteristic curve (AUROC) and a performance of 0.753 AUROC after including five additional predictive features: age, c‐reactive protein (CRP), neutrophil count, estimated glomerular filtration rate (GFR), and albumin [15]. The Royal College of Physicians has endorsed the use of (NEWS2) to predict deterioration of COVID‐19 patients [17]. While NEWS2 have been evaluated, many scores are still not evaluated appropriately, including NEWS, CEWS, MCEWS, AEWS, LDTEWS, and LDTEWS:NEWS. It is critical to evaluate the performance of different available EWS on a COVID‐19 inpatient population, to inform clinical practice during the global pandemic. Improperly validated EWS systems and predictive models are of limited clinical benefit in pandemic settings and their risk of harm can potentially outweigh the promised benefit [18, 19, 20].

In response to the aforementioned clinical and research need to validate EWS in COVID‐19 patients, our work has three main contributions: (i) we evaluate the performance of existing EWS systems (Table 1) that may be currently used in practice to predict clinical deterioration in COVID‐19 patients, (ii) we develop and validate machine learning models to predict deterioration in advance for COVID‐19 patients, and (iii) we compare between machine learning methods and traditional EWS systems.

## 2 MATERIALS AND METHODS

### 2.1 Data source

Deidentified data from patients were obtained from the Infections in Oxfordshire Research Database (IORD) which has Research Ethics Committee, Health Research Authority and Confidentiality Advisory Group approvals (19/SC/0403, ECC5‐017(A)/2009). The dataset includes administrative data, vital sign measurements, laboratory test results and data on the level of oxygen support. We specifically extracted the data of patients who received a positive COVID‐19 diagnosis between 13 March and 30 July, 2020. 2662 patients tested positive for COVID‐19 and 612 of those patients were admitted within a window 48 h prior to positive test to 30 days after. Only the admitted patients were included in the dataset and 101 patients who had a ‘Do Not Resuscitate’ status prior to their COVID test were excluded as their therapy may not have been escalated beyond ward‐based care despite respiratory deterioration. Patients who were immediately escalated to advanced respiratory care upon admission (i.e. within 1 h) were also excluded. The final dataset included 472 patients. Our model features were derived from four sets of commonly collected clinical variables: physiological variables, demographic information, oxygen support level, and laboratory test results (Table 2). The most recent previous blood tests within 5 days of the vital signs observations were considered.

**TABLE 2:** The different feature types and feature sets in our study

**PreVent feature types**	
Feature ype	Features included
Vital signs (F1)	Heart rate, oxygen saturation, respiratory rate, systolic blood pressure, temperature, AVPU
Venous blood tests (F4)	Albumin, ALK. phosphatase, ALT, APTT, basophils, bilirubin creatinine, CRP, eosinophils, haemocrit, haemoglobin, INR lymphocytes, MeanCellVol., monocytes, neutrophils platelets, potassium, prothrombintime, sodium, urea, white cells, eGFR
Blood gas (F5)	BE Act, BloodGas BE Std, Bicarb, Ca+ +, Cl‐ estimated osmolality, FCOHb, glucose, Hb, Hct, K+, MetHb Na+, O${}_{2}$ Sat, cLAC, ctO${}_{2}$c, p5Oc, pCO${}_{2}$ POC, pH, pO${}_{2}$
Variations in vital signs (Var F1)	Mean, max–min, and delta (current value ‐ mean) of vital signs
Delta baseline vital sings (Delta F1)	Current value ‐ historic baseline value from a previous discharge for vital signs. The historic baseline value is extracted from a previous admission. Where a previous admission is missing we imputed with the population mean.
Delta baseline all features (Delta F8)	Current value ‐ historic baseline value from a previous discharge for vital signs, blood tests, and blood gases. The historic baseline value is extracted from a previous admission. Where a previous admission is missing we imputed with the population mean.
**PreVent feature sets**	
Feature set	Features
Vital signs (F1)	HR, RR, SBP, SPO${}_{2}$, TEMP, AVPU, O${}_{2}$ support
EWS bloods (F2)	Albumin, creatinine, haemoglobin, potassium, sodium, urea, white cell count
Vital signs and EWS bloods (F3)	F1 ∪ F2
Venous blood tests (F4)	ALT, albumin, Alk.Phosphatase, basophils, biliru‐bin, CRPCreatinine, eosinophils, haematocrit, haemoglobin, lymphocytes, MeanCellVol monocytes, neutrophils, platelets, potassium, sodium, urea, WhteCell count
Blood gas (F5)	BE ACT, BE STD, BICARB, CA++, CL‐, CLAC, CREAT, CTO${}_{2}$C, estimated osmolality, FCOHB, FHHB, glucose, HB, HCT, K+ METHB, NA+, O${}_{2}$SAT, P5OC, PCO${}_{2}$ POC, PH, PO${}_{2}$, temperature POCT
Venous blood tests and blood gas (F6)	F4 ∪ F5
Vital signs and variations (F7)	F1 ∪ Var F1
All Features (F8)	F1 ∪ F4 ∪ F5
All Features and vital variations (F9)	F8 ∪ Var F1
Vital signs and vital variations and vital baseline delta (F10)	F1 ∪ Var F1 ∪ Delta F1
All features and vital variations and baseline delta (F11)	F8 ∪ Var F1 ∪ Delta F8

#### 2.1.1 Feature sets for EWS systems

The EWS systems (Table 1) assessed each patient for deterioration every time vital signs were measured (NEWS, CEWS, MCEWS, AEWS) or when lab test results were obtained (LDTEWS and LDTEWS:NEWS). Oxygen support was used as a binary predictor for all of the EWS systems, except for LDTEWS and CEWS (in which it was not considered as a predictor in the original publication) [2, 8].

#### 2.1.2 Outcome definition

We defined the outcome of deterioration as either an escalation in the level of oxygen support requirements to either a level 2 or level 3 delivery device, or an unplanned ICU admission within a window of 24 h. To do this, we created four levels of oxygen support based on the respiratory support device used (level 0: room air, level 1: low‐flow oxygen support devices (flow less than 10 L/min, e.g. nasal cannulae), level 2: oxygen support devices with a flow over 10 L/min (e.g. reservoir bag), and level 3: high‐flow ventilation or invasive ventilation). A detailed list of the oxygen support devices used to make the classification is available in Table S5 in Supporting Information. We have defined progression to level 2 or 3 devices as an escalation to high‐level oxygen support; therefore, a patient who progressed from L0 or L1 to L2/L3/unanticipated ICU admission would be considered to have deteriorated, while a transition from L0 to L1 would not be considered a deterioration. L2 indicates a deterioration in the condition of the patient and an increase in the need for respiratory support, while L3 is an advanced level of support that is dependent on respiratory support equipment that is in limited supply (i.e. ventilators or NIV equipment). Outside the scope of this paper, predicting L0 to L1 deterioration can be clinically valuable as it would differentiate patients who can be discharged (patients on room air who do not require oxygen support) from those who need hospital admission (requiring L1 or above support), and a trigger for starting dexamethasone therapy [21, 22]. However, we hypothesised that identifying the patients who need (L3) or are expected to need (L2) the advanced level of support would be more valuable because it provides the clinical teams the opportunity to optimise the management of resources that are in short supply during an event like a pandemic.

### 2.2 Machine learning models

We investigated the performance of (i) a basic machine learning classifier: logistic regression (LR) and (ii) two ensemble learning methods: Random Forest (RF) and Gradient Boosting Trees (GBT). Details of each method, parameter settings, and their strengths and weaknesses are shown in Table S7 in Supporting Information.

#### 2.2.1 Feature sets for machine learning models

To evaluate the EWS performance and compare it with that of the machine learning models, multiple feature sets were considered (Table 2). To ensure fairness, we first established a baseline comparison on the same feature sets as employed by each EWS. For example, the vital signs feature set is used by NEWS, CEWS, MCEWS, AEWS, and hence their performance was compared with a machine learning model using the same input features. Similarly, for the EWS bloods feature set (used by LDTEWS) and the vital signs and EWS bloods feature set (LDTEWS:NEWS), the same inputs were considered for the comparative machine learning models. In addition, we trained and evaluated the machine learning models on various other feature sets. Six sets of clinical parameters were investigated: (1) 24 routinely collected laboratory blood tests, (2) 21 routinely measured/estimated point‐of‐care blood gas readings, (3) changes in vital signs results in a window of 24 h before the given observation, (4) measurements of seven routinely measured physiological parameters, (5) variance of the current vital signs from a baseline of a previous admission, and (6) variance of the current vital signs, blood tests, and blood gases from a baseline of a previous admission. The components of each feature set are detailed in Table 2. Pre‐existing oxygen support before the point of prediction was indicated by a binary variable (1 for L1 support and 0 for L0 support) (Table S5 in Supporting Information). Consequently, we considered the following feature sets for machine learning analysis: (vital signs—F1) vital signs ; (EWS bloods—F2) EWS bloods feature set; (EWS blood and vital signs—F3) a combination of F1 and F2 feature sets; (blood tests—F4) clinical parameters in (1); (blood gas—F5) clinical markers in (2); (blood tests and gas—F6) a combination of F4 and F5 feature sets; (vital signs and delta—F7) a combined feature set of F1 feature set and (3); (all features—F8) a combination of F1, F4, and F5; (all features and delta—F9) a combined feature set of F3, F4, F6; (vital signs and delta baseline and delta—F10) a combination of F1, (3), and (5); and (all features and delta baseline and delta—F11) a combination of F3, F4, F6, and (6).

#### 2.2.2 Calculation of the FiO${}_{2}$ values

Fraction of inspired oxygen (FiO${}_{2}$) values (%) were calculated based on the mask type used. Depending on the mask type, oxygen flow (O${}_{2}$ flow, L/min) and patient's respiratory rate (RR, breaths/min) were included in the calculation. Simple face masks, nebuliser masks, tracheostomy masks and Oxy‐Masks were considered as Hudson masks.

(1) $$\begin{array}{ccc} & & \text{Nasal}\;\text{cannulae:}\;\text{FiO}{}_{2}=(0.038\times O{}_{2}\;\text{flow}+0.208)\times 100[23]\\ & & \text{Hudson}\;\text{mask:}\;\text{FiO}{}_{2}=-0.99\times \text{RR}+3.11\times O{}_{2}\;\text{flow}\\ & & \;\;\;\;\;\;+\;51.05[24]\\ & & \text{Non-rebreather}\;\text{mask:}\;\text{FiO}{}_{2}=80[25]\\ & & \text{Face}\;\text{mask}\;\text{with}\;\text{reservoir:}\;\text{FiO}{}_{2}=80[25]\\ & & \text{Venturi}\;\text{mask:}\;\text{FiO}{}_{2}=21,24,28,35,40,60\\ & & \;\text{(depending}\;\text{on}\;\mathrm{model)}\\ & & \text{Room}\;\text{air:}\;\text{FiO}{}_{2}=21\\ & & \text{CPAP}\;\text{and}\;\text{other}\;\text{non-invasive}\;\text{systems:}\;\text{FiO}{}_{2}=100 \end{array}$$

#### 2.2.3 Data preprocessing

We treated observation sets as independent rather than as grouped by patient admission. We excluded implausible physiological values. Non‐numerical readings were replaced with clinically appropriate values. Where a lab value was reported as being below the threshold of detection of the laboratory assay, the value was replaced with a numerical zero value. Where values were reported as being above the threshold of detection, clinically appropriate values were selected to maintain the significance of the high result. When the provision or absence of supplemental oxygen was missing, we assumed that supplemental oxygen was not provided and set the supplemental oxygen value to 0. Similarly, we have made the same assumption for AVPU and replaced missing AVPU values by ‘alert’. For missing values, we have used multiple imputation techniques (mean, median, Bayesian ridge regression, and stochastic regression) to compensate for missing values across the dataset. The best performing imputation method across different experiments was median, hence we have chosen it as the default method in our analysis as a design choice (Figure S1 in Supporting Information).

#### 2.2.4 Alerting thresholds

The evaluated EWS systems (Table 3) are provided with default alerting thresholds to convert the computed score to ‘alert’ or ‘no alert’. NEWS gives an individual score of 2 when a patient is on supplementary oxygen, and an individual score of 0 when the patient is on room air. NEWS aggregates the individual scores to an overall score which is assessed against an alerting threshold (default 5). We used the default individual scores for EWS. However, given that we are predicting an outcome different from the default predicted outcome of the EWS (escalation in oxygen demand vs. in‐hospital mortality, cardiac arrest or unplanned ICU admission), we chose to evaluate the performance of the EWS not only based on the original overall thresholds (e.g. 5 for NEWS) but also on optimised thresholds.

**TABLE 3:** This table highlights the performance of the Early Warning Scores. Section A of the table describes the performance of the original thresholds for the Early Warning Scores. NEWS has two recommended thresholds (5 and 7) and LDTEWS:NEWS also has two recommended thresholds (0.27 and 0.36). We measured the performance of 5 and 0.27 for NEWS and LDTEWS:NEWS respectively as those are the most commonly used thresholds. Section B outlines the performance of the accuracy‐optimised thresholds

**The performance of the original thresholds (Section A)**						
**Score**	**Threshold**	**Acc**	**Sen**	**Sps**	**Prs**	**AUROC**
NEWS	5	0.75 (0.75–0.75)	0.66 (0.65–0.68)	0.75 (0.75–0.75)	0.02 (0.02–0.02)	0.79 (0.78–0.79)
CEWS	4	0.91 (0.91–0.91)	0.23 (0.21–0.24)	0.91 (0.91–0.91)	0.02 (0.02–0.02)	0.63 (0.61–0.64)
MCEWS	4	0.78 (0.78–0.78)	0.61 (0.59–0.62)	0.78 (0.78–0.78)	0.02 (0.02–0.02)	0.78 (0.77–0.79)
LDTEWS	0.33	0.73 (0.73–0.73)	0.41 (0.40–0.43)	0.74 (0.73–0.74)	0.01 (0.01–0.01)	0.62 (0.61–0.63)
LDTEWS:NEWS	0.27	0.84 (0.84–0.84)	0.52 (0.50–0.53)	0.84 (0.84–0.85)	0.03 (0.03–0.03)	0.80 (0.79–0.80)
**The performance of the accuracy‐optimised thresholds (Section B)**						
**Score**	**Threshold**	**Acc**	**Sen**	**Sps**	**Prs**	**AUROC**
NEWS	4	0.62 (0.60–0.64)	0.77 (0.75–0.79)	0.62 (0.60–0.64)	0.02 (0.02–0.02)	0.79 (0.78–0.79)
CEWS	2	0.64 (0.62–0.65)	0.55 (0.52–0.57)	0.64 (0.62–0.65)	0.01 (0.01–0.01)	0.63 (0.61–0.64)
MCEWS	3	0.61 (0.59–0.63)	0.76 (0.74–0.79)	0.61 (0.59–0.62)	0.02 (0.02–0.02)	0.78 (0.77–0.79)
AEWS	4	0.60 (0.59–0.62)	0.66 (0.64–0.68)	0.60 (0.59–0.62)	0.01 (0.01–0.01)	0.68 (0.67–0.69)
LDTEWS	0.18 (0.17–0.18)	0.52 (0.52–0.53)	0.75 (0.74–0.76)	0.52 (0.52–0.53)	0.01 (0.01–0.01)	0.62 (0.61–0.63)
LDTEWS:NEWS	0.21 (0.20–0.21)	0.67 (0.66–0.68)	0.77 (0.76–0.79)	0.67 (0.66–0.68)	0.02 (0.02–0.02)	0.80 (0.79–0.80)

We optimised the machine learning and EWS thresholds to report the performance metrics on the test set by identifying the thresholds that maximise the accuracy on the train dataset, and used these thresholds on the test set.

### 2.3 Performance assessment

For all experiments, the classification was performed by training on a balanced dataset and then testing on an imbalanced (representative) dataset. We ran the classification over multiple iterations and cross‐fold validations. In each fold, 20% of the data was considered as the test set. Within the remaining 80% of the data, since non‐events outnumbered events, non‐events were sub‐sampled randomly to balance the size of the two classes. This was run over 40 iterations of 5‐fold stratified cross‐validation. We chose *k*‐fold stratified due to the imbalanced nature of the classes.

For machine learning, GBT, RF, and LR were considered as basic machine learning techniques (Table S7 in Supporting Information). For EWS, the test set was used to calculate EWS scores in each fold.

We evaluated the performance of our EWS and machine learning methods using the AUROC to predict an outcome of deterioration defined as either escalation in the level of oxygen demand (to level 2 or 3) (Table S5 in Supporting Information) or an unplanned ICU admission. The performance in terms of accuracy, sensitivity, specificity, precision, and AUROC were calculated for the validation sets (for parameter setting) and test sets (for final comparison) and averaged over iterations; mean and standard deviation were reported.

(2) $$\begin{array}{cc} & \text{Accuracy}=\frac{\text{TP}\;+\;\text{TN}}{\text{TP}\;+\;\text{TN}\;+\;\text{FP}\;+\;\text{FN}}\\ & \text{Sensitivity}=\frac{\text{TP}}{\text{TP}\;+\;\text{FN}}\\ & \text{Specificity}=\frac{\text{TN}}{\text{TN}\;+\;\text{FP}}\\ & \text{Precision}=\frac{\text{TP}}{\text{TP}\;+\;\text{FP}}\\ & \text{F1-score}=2\frac{\text{Precision}\times \text{Sensitivity}}{\text{Precision}\;+\;\text{Sensitivity}} \end{array}$$

TP, TN, FP, and FN stand for true positive, true negative, false positive, and false negative, respectively. Considering a probability estimate as the output of each classifier for the validation set and setting various thresholds to categorise this output as event/non‐event could result in different TP, FP, FN, and TN rates. Alternatively, a receiver operating characteristic (ROC) curve showing the sensitivity as a function of 1—specificity for different thresholds; each point in the curve indicates a specific value for sensitivity, specificity, and accuracy. AUROC‐ROC is the area under the ROC curve.

The parameters of the models (e.g. number of ensembles for RF or GBT) were optimised through the internal cross‐validation on the training data. This was done by a grid search over a range of values and selecting parameters that generated the best AUROC‐ROC. The model with the highest performance was reported in the paper.

### 2.4 Patient and public involvement

The IORD panel, which includes patient and public representatives provided feedback on the study design and approved its final form.

## 3 RESULTS

### 3.1 Patient cohort and features characteristics

Our study is retrospective, using data extracted from electronic health records (EHR). The dataset contains routinely collected observations from concluded hospital admissions from four hospitals within the Oxford University Hospitals (OUH) NHS Foundation Trust. OUH consists of 4 teaching hospitals in Oxfordshire, UK, serving a population of 600,000 and providing tertiary referral services to the surrounding region. Data were obtained between March and July 2020.

Our dataset included 15,686 sets of observations from 472 admissions in 472 unique patients. The dataset included 137 respiratory deterioration events (observing each patient until discharge or their first deterioration). The average age was 68 ± 16 (mean ± std) and 47% (221/472 patients) of the dataset were females. The mean and interquartile ranges (IQR) of the blood, blood gas, and vital sign parameters are in the supplementary materials (Tables S2–S4 in Supporting Information).

### 3.2 Performance evaluation

#### 3.2.1 Outcome definition

We defined respiratory deterioration as the need for advanced respiratory support (high‐flow nasal oxygen [HFN0], continuous positive airways pressure [CPAP], NIV(intubation) or ICU admission within a prediction window of 24 h. It should be noted, however, that hypoxic respiratory failure is not the only process through which COVID‐19 patients deteriorate as some patients deteriorate through a process of shock due to venous thromboembolism or super‐added sepsis. Such events may also lead to ICU admission or increased oxygen requirements and so still be captured by our model.

#### 3.2.2 Performance of the EWS systems

**FIGURE 1:**
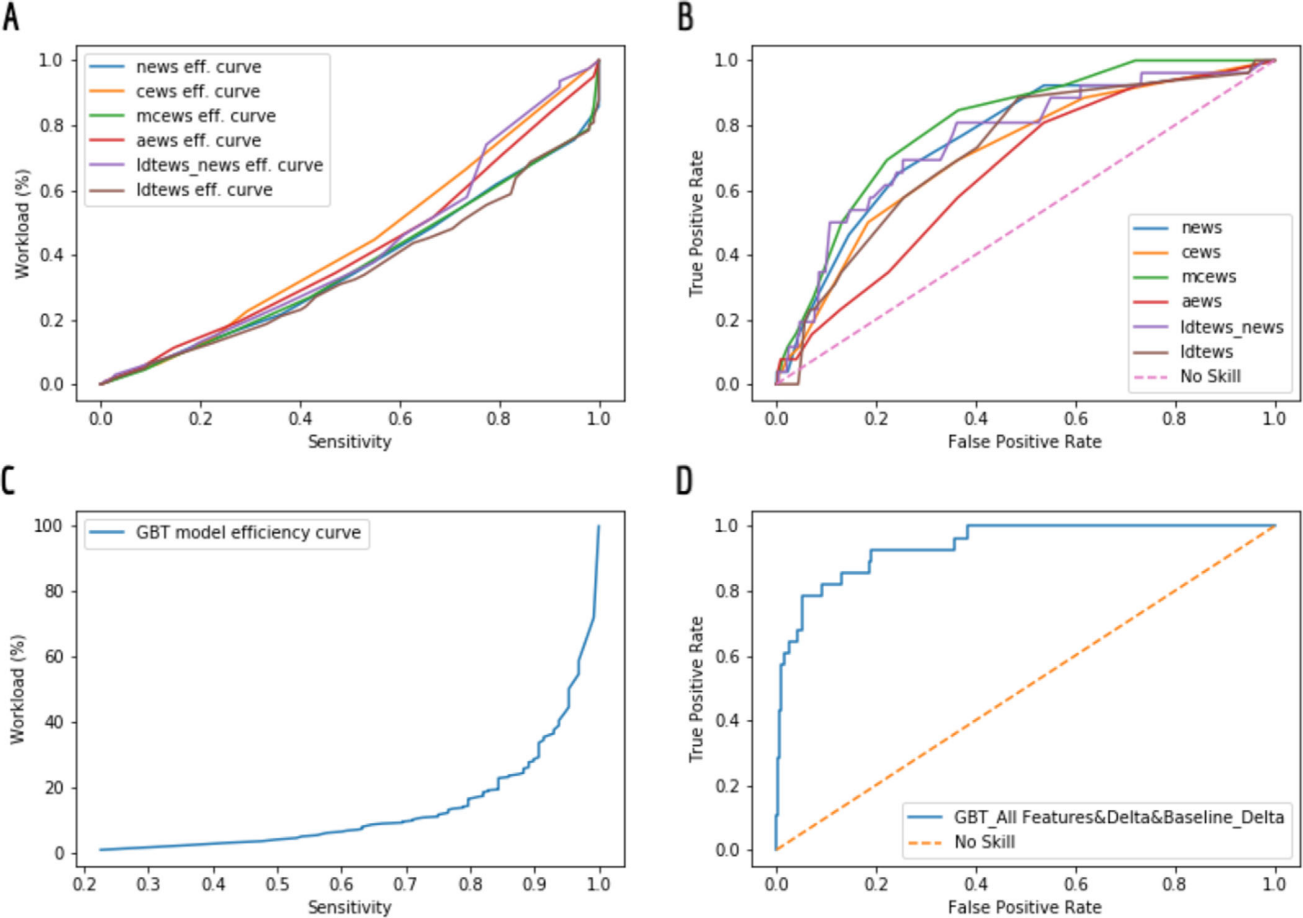
This figure includes the efficiency and Receiver Operating Characteristic (ROC) curves for the machine learning models and the Early Warning Scores (EWS). (a) The efficiency curves for the EWS in our study. The low performance of the EWS on the Efficiency Curve metric may be explained by a high false positive. (b) The ROC curves for the various EWS in our study (the best performance is for NEWS with AUROC of 72%). (C) The performance of the GBT model measured by the efficiency curve metric. (d) The ROC and AUROC for the GBT model on F9 feature set (AUROC of 94%)

Table 3 outlines the performance of the EWS systems. NEWS, MCEWS, CEWS, AEWS, LDTEWS:NEWS, and LDTEWS achieved an AUROC of 79%, 78%, 63%, 68%, 80%, 62%, respectively. The best performing scores were NEWS and LDTEWS:NEWS (Figure 1). The efficiency curve of the various EWS systems is outlined in Figure 1.

We evaluated the performance of the recommended (original) thresholds for the different EWS. The default thresholds are 5, 4, 4, 0.27, 0.33 for NEWS, CEWS, MCEWS, LDTEWS:NEWS, and LDTEWS, respectively. AEWS does not have a recommended threshold, therefore we have excluded it from the evaluation of the recommended thresholds. The NEWS score had the most balanced sensitivity and specificity (66% and 75%, respectively). NEWS and LDTEWS achieved the lowest accuracy (75% and 73%) with a sensitivity and specificity of 41% and 74% for LDTEWS. CEWS achieved the highest accuracy (91%) but with a sensitivity of 23% and specificity of 91% (Table 3).

We optimised the thresholds for each score to maximise accuracy as outlined in the Methods Section. Optimised EWS thresholds yielded more balanced performance. LDTEWS:NEWS was the overall best performing score with an accuracy, sensitivity, and specificity of 67%, 77% and 67%, respectively. NEWS, MCEWS, CEWS, and AEWS achieved high accuracy (62%, 61%, 64%, 60%, respectively). The worst performing score was LDTEWS with an accuracy of 52% and AUROC of 62%. The accuracy‐optimised thresholds for all scores differed from the recommended values (Table 3).

The performance of the EWS in COVID‐19 patients was significantly lower than that previously reported in non‐COVID patients. The Royal College of Physicians [6] reported a performance of (AUROC = 89%) for NEWS compared to (AUROC = 79%) in our dataset. Watkinson and colleagues [3] reported a performance of (AUROC = 86.8% and AUROC = 80.8%) for MCEWS and CEWS, respectively. This compares to AUROC values of 78% and 63% for MCEWS and CEWS in our dataset. Shamout and colleagues [7] reported that AEWS achieved an AUROC of 83.8%, while AEWS achieved a performance of 68% on COVID patients in our dataset. Redfern and colleagues reported an AUROC of 90.1–91.6% for LDTEWS:NEWS. In COVID patients, the AUROC for LDTEWS:NEWS was 80%. The worst performing score in our study was LDTEWS (AUROC of 62%). The score was developed by Jarvis and colleagues [8] with a reported AUROC that ranges between 75% and 80% in discriminating in‐hospital mortality among the general in‐hospital patient cohort. This indicates that while the predictors used in LDTEWS (HGB, Alb, Na, k, Cr, Ur, WBC) are useful to discriminate in‐hospital mortality in non‐COVID, they are less useful in predicting respiratory deterioration in COVID patients (Table 1).

#### 3.2.3 Performance of the machine learning models

We evaluated the performance of three machine learning models (GBT, RF, and LR) on the training data using an internal 5‐fold cross‐validation. We evaluated the performance of the machine learning models on multiple feature sets as outlined in the feature sets subsection of the Methods and Table 2 (F1–F11). The GBT model outperformed the other models on the different features sets in our training dataset. Therefore, we made a design choice to use only the GBT model when evaluating the performance on the different feature sets in the test data. The highest AUROC was achieved using the F1 (AUROC of 83%), F7 (AUROC of 93%), F8 (AUROC of 86%), F9 (AUROC of 94%), and F11 (AUROC of 93%) feature sets. The lowest AUROC was observed in the F2 (AUROC of 72%), F4 (AUROC of 77%), F5 (AUROC of 69%), and F6 (AUROC of 78%) feature sets. The F7 dataset is a simple feature set that is based on 6 commonly collected vital signs and their variability. F7 could represent the scenario of an overrun healthcare facility in which access to lab tests may not be easily accessible and readily available.

We compared the performance of the EWS systems and machine learning models to predict COVID‐19 patient deterioration in three main feature sets: F1–F3 (Table 2). In each of the three feature sets, the machine learning model outperformed the EWS systems. For the F1 feature set, we can compare the performance of NEWS (AUROC = 79%), MCEWS (AUROC = 78%), CEWS (AUROC = 63%), and AEWS (AUROC = 68%) with the performance of GBT (AUROC = 83%). For the F2 feature set, we can compare the performance of LDTEWS (AUROC = 62%) with the performance of GBT (AUROC = 72%). For the F3 set, we can compare the performance of LDTEWS:NEWS (AUROC = 80%) with the performance of GBT (AUROC = 85%) (Figure 1). The efficiency curve of machine learning EWS systems is outlined in Figure 1.

The overall best performing algorithm for machine learning models was the GBT model on the F9 feature set (AUROC = 94%). Given the imbalanced nature of our dataset, we have decided to tune the probability‐class conversion threshold for the GBT model to create the best performing machine learning model. We decided to optimise the threshold to maximise accuracy. We identified the threshold that maximises the accuracy of the GBT model on the training set and measured the performance on the test set. The identified threshold was 0.19. The optimised GBT model achieved an accuracy, sensitivity, and specificity of 70%, 96%, and 70%, respectively. The most and least important features are outlined in Table 4. Out of the 10 most important features (FiO${}_{2}$, min–max SBP, CRP, max–min HR, PO${}_{2}$, mean cell volume, arterial blood calcium, max–min RR, CtO${}_{2}$C, temp), four belonged to the F7 (vital signs and variability) feature set, three belonged to the F5 feature set (arterial blood tests), and two belonged to the F4 feature set (venous blood tests). The most important feature was FiO${}_{2}$. Delta is a measure of variability of a specific variable, it is calculated as (current value—the mean in the last 24 h). The most important vital signs were heart rate, respiratory rate, temperature, and blood oxygen saturation (SpO${}_{2}$).

**TABLE 4:** The performance of machine learning models and the corresponding feature weights for the most and least important features; Sections A, B, C, and D explore the performance of the best machine learning model (GBT model on the F9 feature set). Section A outlines the performance of the accuracy‐optimised threshold for the GBT model. The threshold was set on the training data and tested on the test data. Section B outlines the performance of the GBT model after limiting the predictors to the 20 most important features. Sections C and D outline the performance of the GBT model after reducing the look back window from 24 h to 6 and 12 h. Section E outlines the performance after adding FiO_2_ as a predictor. FiO_2_ did not improve model performance compared to the optimised threshold model (section A of table 4); however, it ranked as the most important feature on feature importance analysis. Section F outlines the performance of the model after adding age as a predictor. Age did not rank within the most important features and did not improve performance over the performance of the optimised threshold model (section A of table 4). Section G outlines the performance after adding delta baseline to the vital signs and variations feature set. Section H outlines the performance after adding delta baseline to the all features and vital variations feature set. Section I outlines the performance of the GBT model with the hard output threshold on the different feature spaces before identifying the best model and adjusting the threshold for accuracy. Section J outlines the feature importance for the GBT model on the F9 feature set. It includes the nine most and least important features

**Section**	**Feature**	**Model**	**Threshold (on train)**	**Acc**	**Sen**	**Sps**	**Prs**	**AUROC**
A: Accuracy‐optimised threshold	F9	GBT	0.12 (0.11–0.13)	0.70 (0.69–0.71)	0.96 (0.95–0.96)	0.70 (0.69–0.70)	0.03 (0.03–0.03)	0.94 (0.94–0.94)
B: Feature selection	F9 (top 20)	GBT	0.35 (0.32–0.37)	0.80 (0.80–0.81)	0.91 (0.90–0.92)	0.80 (0.80–0.81)	0.04 (0.04–0.04)	0.94 (0.94–0.94)
C: 6‐h lookback window	F9	GBT	0.09 (0.08–0.09)	0.56 (0.56–0.57)	0.87 (0.86–0.88)	0.56 (0.56–0.56)	0.01 (0.01–0.01)	0.85 (0.85–0.85)
D: 12‐h lookback window	F9	GBT	0.32 (0.30–0.34)	0.66 (0.65–0.67)	0.87 (0.86–0.88)	0.66 (0.65–0.67)	0.02 (0.02–0.02)	0.86 (0.86–0.86)
E: Adding FiO_2_ as a predictor	F9 and FiO_2_	GBT	0.15 (0.13–0.18)	0.72 (0.71–0.73)	0.89 (0.87–0.91)	0.72 (0.71–0.73)	0.03 (0.03–0.03)	0.93 (0.93–0.93)
F: Adding Age as a predictor	F9 and age	GBT	0.19 (0.17–0.21)	0.73 (0.72–0.74)	0.94 (0.94–0.95)	0.73 (0.72–0.74)	0.03 (0.03–0.03)	0.93 (0.93–0.94)
G: Adding Delta baseline to vital signs and delta	F10	GBT	0.32 (0.30–0.34)	0.82 (0.81–0.83)	0.87 (0.86–0.88)	0.82 (0.81–0.83)	0.04 (0.04–0.04)	0.93 (0.92–0.93)
H: Adding Delta baseline to all features and delta	F11	GBT	0.22 (0.21–0.24)	0.74 (0.73–0.74)	0.93 (0.92–0.93)	0.73 (0.73–0.74)	0.03 (0.03–0.03)	0.93 (0.92–0.93)
**Hard output performance (Section I)**								
**Feature**	**Model**	**AUROC**						
F1	GBT	0.83 (0.83–0.84)						
F2	GBT	0.72 (0.71–0.72)						
F3	GBT	0.85 (0.84–0.85)						
F4	GBT	0.77 (0.76–0.77)						
F5	GBT	0.69 (0.68–0.69)						
F6	GBT	0.78 (0.78–0.79)						
F7	GBT	0.93 (0.92–0.93)						
F8	GBT	0.86 (0.86–0.87)						
F9	GBT	0.94 (0.94–0.94)						
F10	GBT	0.93 (0.92–0.93)						
F11	GBT	0.93 (0.93–0.93)						
**Feature weights (Section J)**								
**Highest feature weights**		**Lowest feature weights**						
FiO_2_	0.258354	Bilirubin‐umol/L	0.000031					
Max‐Min SBP	0.151461	METHB (BG)	0.000020					
CRP‐mg/L	0.108911	FCOHB (BG)	0.000011					
Max‐Min HR	0.044093	CLAC (BG)	0.000009					
PO_2_ (BG)	0.033090	NA+ (BG)	0.000007					
Mean CellVol‐fL	0.026848	Basophils‐x10^9^/L	0.000003					
CA+ + (BG)	0.026313	masktyp	0					
Max‐Min RR	0.025169	TEMPERATURE POCT	0					
CTO_2_C (BG)	0.024777	Potassium‐mmol/L	0					
TEMP	0.021500	avpu	0					

We conducted three additional experiments. The first was to limit the predictors of the GBT model to the top features that ranked the highest on the feature importance scale considering the training set. We found that the optimal number of features was 18–20 and subsequently chose to report the performance on the 20 most important features. This forward selection experiment did not impact performance (Table 4). We did not attempt a backward selection approach in this study, which is considered preferable in classical statistics. The second experiment was to include a more granular measurement of oxygen support. We included the Fraction of Inspired Oxygen (FiO${}_{2}$) for this aim. Including the FiO${}_{2}$ did not improve the performance (Table 4). The third experiment was to include age as a predictor. Including age as a predictor did not significantly impact the performance (Table 4). The lack of performance gains in spite of the high feature importance may be due to multicolinearity, where a subset of existing variables highly correlate with this feature. This is explicit in the construction of the FiO${}_{2}$ variable, which is calculated from source variables already present in the vital signs feature set (respiratory rate, SpO${}_{2}$, Masktype) as outlined in the Methods section.

Our results show that summary measures of variability of vital signs and laboratory markers play an important role in predicting deterioration. Adding the variability (range, mean of previous 24‐h window) and delta (current value ‐ mean) features to the vital signs feature set added 10% points to the AUROC (vital signs 83% vs. vital signs and variations 93%). Similar results were observed in the all features feature set, where adding the variability and delta predictors added 8% points to AUROC (all features 86% vs. all features and variations 94%). Adding the delta baseline variables to both the all feature and vital signs feature spaces has improved the performance (vital signs and variations and baseline 93%; all features and variations and baseline 93%). These observations echo common clinical practice where physicians often analyse trends of parameters rather than their absolute values when evaluating a patient and highlight the benefits of dynamic monitoring. Moreover, the importance of summarising the variability and changes of vital signs when using them as inputs for machine learning models has already been demonstrated by Shamout and colleagues [26] in their work to develop a deep learning‐based early warning system.

The lower performance of the model when using variables from blood gas analysis could partly be explained by inconsistency in the labelling of these samples. The origin of the blood, whether venous or arterial, was frequently missing or mislabelled perhaps reflecting time pressures on clinical staff, or skewed where interest is towards markers minimally influenced by sample provenance (e.g. lactate). This required the use of imputation techniques during the preprocessing of the dataset, which may have had an effect on performance. Moreover, some data points in blood gas readings duplicated information encoded within other feature sets, such as haemoglobin and creatinine.

### 3.3 Classification and misclassification

To assess for biases in model performance, we assessed rates of patient misclassification during validation for the best performing machine learning technique. We observed that rates of misclassification were higher for white (44%) than black, Asian and minority ethnic group patients (22%). The misclassification rate was similar between men (47%) and women (42%) and between patients aged over 60 (43%) and patients aged between 18 and 60 (44%). The difference in performance may be explained by feature differences across ethnicity groups including genetic and blood biomarkers.

## 4 DISCUSSION

Here, we assess the performance of existing EWS for predicting escalation to high‐level oxygen support or unplanned ICU admission; this is an area of clinical importance in COVID‐19. The EWS studied have been previously validated for predicting events such as cardiac arrest, unplanned ICU admission or death (Table 1). However, limitations of using death as an outcome measure include that the score may be identifying an early sign of an already irreversible process, and therefore early identification of this may offer limited opportunity for clinical intervention. By contrast, our COVID‐19 focused outcome measure provides a clinically useful and actionable warning which may help clinicians and healthcare system managers to preempt shortages and optimise resource allocation in a pandemic context. The difference in performance between our model and the EWS could be partially explained by the fact that these EWS were developed and optimised to detect ward patients' deterioration against different outcomes. Nonetheless, these EWS represent the current standard of care for COVID‐19 patients, and we took action to mitigate these effects by optimising each EWS threshold for our COVID‐19 inpatient cohort. The alerting threshold for EWS systems should be optimised according to the requirements of the clinical settings, taking into account that sensitivity and specificity are usually inversely correlated and that high false alert rate (calculated as False positive rate = 1 ‐ specificity) leads to alert ‘fatigue’ and inefficient use of clinical resources. Within the prediction window of 24 h (Table 4), our machine learning EWS system achieves a specificity that ranges between 70% to 82%, which means a false positive rate of 30% to 18%. Future iterations of the system will aim to increase specificity by including comorbidities, using transfer learning, considering a larger dataset, and employing more advanced models.

A strength of our machine learning approach is its interpretability, using methods employed elsewhere in clinical practice [27] and shown able to attain patient and clinician trust. The three selected models (GBT, RF, and LR) permit querying of variables' weights and presentation in an explainable way. This ability to make sense of the algorithm decision‐making process has repeatedly been described as a critical factor in increasing technology uptake in clinical practice [28]. Moreover, our feature sets are oriented around routinely collected clinical data collected within existing care pathways, including calculation of EWS scores. Our models are therefore rapidly deployable within current clinical pathways.

A relative limitation of our study is that the number of features approximates that of patients within the training set. There is consequently a risk of overfitting when considering all the clinical features available, as exemplified by the increase in performance when limiting our inputs to the 20 most influential variables. Additionally, while a prediction window >1 h before an event is in line with existing vital monitoring systems [27], the window between sets of vital signs and positive events may capture overlapping transition effects. For example, an escalation in FiO${}_{2}$ may represent an emergency response to physiological deterioration which is necessarily followed by escalation of oxygen delivery device and ICU admission (where that level of support can be provided). Therefore, predictions made after a rapid escalation in FiO${}_{2}$ may capture patients where a clinical deterioration has already occurred, and ICU admission/higher level respiratory support is presently being arranged. Increasing the window upper bound to 3 h before an escalation event had minimal effect on performance. On examination, the majority of escalation events (109/137) contained observations within a 12–24 h prior window. Therefore, our data suggests that this is not a significant limitation in our case. Another limitation of our study is that we have not included pre‐existing conditions in our analysis. Pre‐existing conditions have shown to play an important role in increasing the risk of COVID‐19 deterioration. Therefore, future research efforts should include a pre‐existing conditions as predictive features in COVID‐19 predictive models. Moreover, while we hypothesise that multicolinearity could explain why the addition of FiO${}_{2}$ did not increase AUROC, we did not explore multicolinearity between the different features in our dataset. This could be a valuable piece of analysis to explore in future research efforts.

The multivariate nature of EWS permit partial scores to be calculated where data is missing, however the machine learning methods examined require prior handling of missing data. These can be challenging to impute, as clinical data is often not missing at random. Missing data may therefore be poorly represented by population average values; for example, recording of vital signs is performed less frequently where there is no clinical concern. Limitations of imputation strategies include also the loss of important metadata. The presence of a measurement can often encode clinical meaning, for example, the presence of an arterial blood gas reading is often driven by clinical concern of respiratory compromise; semantic knowledge which is lost by imputation. Nonetheless, in this study we demonstrate minimal difference in model performance across a range of imputation strategies on model performances, demonstrating minimal difference. By contrast, the multivariate, interpretable nature of EWS permit a partial score to be calculate despite missingness.

## 5 CONCLUSION

Here, we assessed the theoretical performance of three machine learning approaches against some of the current EWS. We demonstrated that the performance of EWS in COVID‐19 patients is sub‐optimal. We also present a machine learning Early Warning System with AUROC of 94%. Translation to clinical practice requires further optimisation and prospective valuation in a representative clinical population. Such optimisations include a better understanding of the dynamic evolution and availability of clinical data in real time in the healthcare setting. Calibration of alarm trigger thresholds should be guided by desired clinical performance, reflecting healthcare system resource constraints and priorities, and a product design accounting for an optimal human–computer interaction.

## Supporting information

Supporting Information

## References

[1] Morgan R.J.M., Wright M.M. In defence of early warning scores. British Journal of Anaesthesia 99, (5), 747–748 (2007). 10.1093/bja/aem286 17933804

[2] Tarassenko Lionel, Clifton David A., Pinsky Michael R., Hravnak Marilyn T., Woods John R., Watkinson Peter J. Centile‐based early warning scores derived from statistical distributions of vital signs. Resuscitation 82, (8), 1013–1018 (2011). 10.1016/j.resuscitation.2011.03.006 21482011

[3] Watkinson Peter J., Pimentel Marco A.F., Clifton David A., Tarassenko Lionel Manual centile‐based early warning scores derived from statistical distributions of observational vital‐sign data. Resuscitation 129, 55–60 (2018). 10.1016/j.resuscitation.2018.06.003 29879432 PMC6062656

[4] Redfern Oliver C., Pimentel Marco A.F., Prytherch David, Meredith Paul, Clifton David A., Tarassenko Lionel, Smith Gary B., Watkinson Peter J. Predicting in‐hospital mortality and unanticipated admissions to the intensive care unit using routinely collected blood tests and vital signs: Development and validation of a multivariable model. Resuscitation 133, 75–81 (2018). 10.1016/j.resuscitation.2018.09.021 30253229 PMC6562198

[5] Pimentel Marco A.F., Redfern Oliver C., Gerry Stephen, Collins Gary S., Malycha James, Prytherch David, Schmidt Paul E., Smith Gary B., Watkinson Peter J. A comparison of the ability of the National Early Warning Score and the National Early Warning Score 2 to identify patients at risk of in‐hospital mortality: A multi‐centre database study. Resuscitation 134, 147–156 (2019). 10.1016/j.resuscitation.2018.09.026 30287355 PMC6995996

[6] Jones Mike NEWSDIG: The National Early Warning Score Development and Implementation Group. Clinical Medicine 12, (6), 501–503 (2012). 10.7861/clinmedicine.12-6-501 23342400 PMC5922584

[7] Shamout Farah, Zhu Tingting, Clifton Lei, Briggs Jim, Prytherch David, Meredith Paul, Tarassenko Lionel, Watkinson Peter J, Clifton David A Early warning score adjusted for age to predict the composite outcome of mortality, cardiac arrest or unplanned intensive care unit admission using observational vital‐sign data: a multicentre development and validation. BMJ Open 9, (11), e033301 (2019). 10.1136/bmjopen-2019-033301 PMC6887005 31748313

[8] Jarvis Stuart W., Kovacs Caroline, Badriyah Tessy, Briggs Jim, Mohammed Mohammed A., Meredith Paul, Schmidt Paul E., Featherstone Peter I., Prytherch David R., Smith Gary B. Development and validation of a decision tree early warning score based on routine laboratory test results for the discrimination of hospital mortality in emergency medical admissions. Resuscitation 84, (11), 1494–1499 (2013). 10.1016/j.resuscitation.2013.05.018 23732049

[9] Chen Tao, Wu Di, Chen Huilong, Yan Weiming, Yang Danlei, Chen Guang, Ma Ke, Xu Dong, Yu Haijing, Wang Hongwu, Wang Tao, Guo Wei, Chen Jia, Ding Chen, Zhang Xiaoping, Huang Jiaquan, Han Meifang, Li Shusheng, Luo Xiaoping, Zhao Jianping, Ning Qin Clinical characteristics of 113 deceased patients with coronavirus disease 2019: retrospective study. BMJm1091(2020). 10.1136/bmj.m1091 32217556 PMC7190011

[10] Zou Xiaojing, Li Shusheng, Fang Minghao, Hu Ming, Bian Yi, Ling Jianmin, Yu Shanshan, Jing Liang, Li Donghui, Huang Jiao Acute Physiology and Chronic Health Evaluation II Score as a Predictor of Hospital Mortality in Patients of Coronavirus Disease 2019. Critical Care Medicine 48, (8), e657–e665 (2020). 10.1097/ccm.0000000000004411 32697506 PMC7217128

[11] Ihle‐Hansen Håkon, Berge Trygve, Tveita Anders, Rønning Else Johanne, Ernø Per Erik, Andersen Elizabeth Lyster, Wang Christian Hjorth, Tveit Arnljot, Myrstad Marius Covid‐19: Symptomer, forløp og bruk av kliniske skåringsverktøy hos de 42 første pasientene innlagt på et norsk lokalsykehus. Tidsskrift for Den norske legeforening(2020). 10.4045/tidsskr.20.0301 32378844

[12] Semeraro Federico, Scquizzato Tommaso, Scapigliati Andrea, Ristagno Giuseppe, Gamberini Lorenzo, Tartaglione Marco, Dell’Arciprete Oscar, Mora Fabio, Cordenons Fiorella, Del Giudice Donatella, Picoco Cosimo, Gordini Giovanni New Early Warning Score: off‐label approach for Covid‐19 outbreak patient deterioration in the community. Resuscitation 151, 24–25 (2020). 10.1016/j.resuscitation.2020.04.018 32325095 PMC7169898

[13] Hu Hai, Yao Ni, Qiu Yanru Comparing Rapid Scoring Systems in Mortality Prediction of Critically Ill Patients With Novel Coronavirus Disease. Academic Emergency Medicine 27, (6), 461–468 (2020). 10.1111/acem.13992 32311790 PMC7264631

[14] Stow Daniel, Barker Robert, Matthews Fiona, Hanratty Barbara National Early Warning Scores And COVID‐19 Deaths In Care Homes: An Ecological Time Series Study. Innovation in Aging 4, (Supplement_1), 962–963 (2020). 10.1093/geroni/igaa057.3517 PMC8438578 34518247

[15] Carr Ewan, Bendayan Rebecca, Bean Daniel, Stammers Matt, Wang Wenjuan, Zhang Huayu, Searle Thomas, Kraljevic Zeljko, Shek Anthony, Phan Hang T. T., Muruet Walter, Gupta Rishi K., Shinton Anthony J., Wyatt Mike, Shi Ting, Zhang Xin, Pickles Andrew, Stahl Daniel, Zakeri Rosita, Noursadeghi Mahdad, O’Gallagher Kevin, Rogers Matt, Folarin Amos, Karwath Andreas, Wickstrøm Kristin E., Köhn‐Luque Alvaro, Slater Luke, Cardoso Victor Roth, Bourdeaux Christopher, Holten Aleksander Rygh, Ball Simon, McWilliams Chris, Roguski Lukasz, Borca Florina, Batchelor James, Amundsen Erik Koldberg, Wu Xiaodong, Gkoutos Georgios V., Sun Jiaxing, Pinto Ashwin, Guthrie Bruce, Breen Cormac, Douiri Abdel, Wu Honghan, Curcin Vasa, Teo James T., Shah Ajay M., Dobson Richard J. B. Evaluation and improvement of the National Early Warning Score (NEWS2) for COVID‐19: a multi‐hospital study. BMC Medicine 19, (1), (2021). 10.1186/s12916-020-01893-3 PMC7817348 33472631

[16] Meylan Sylvain, Akrour Rachid, Regina Jean, Bart Pierre‐Alexandre, Dami Fabrice, Calandra Thierry An Early Warning Score to predict ICU admission in COVID‐19 positive patients. Journal of Infection 81, (5), 816–846 (2020). 10.1016/j.jinf.2020.05.047 PMC7263266 32474038

[17] Royal College of Physicians (RCP) . NEWS2 and deterioration in COVID‐19; (2020). Available at: https://www.rcplondon.ac.uk/news/news2-and-deterioration-covid-19 (Accessed: 05 April 2020)

[18] Wynants Laure, Van Calster Ben, Collins Gary S, Riley Richard D, Heinze Georg, Schuit Ewoud, Bonten Marc M J, Dahly Darren L, Damen Johanna A, Debray Thomas P A, de Jong Valentijn M T, De Vos Maarten, Dhiman Paula, Haller Maria C, Harhay Michael O, Henckaerts Liesbet, Heus Pauline, Kammer Michael, Kreuzberger Nina, Lohmann Anna, Luijken Kim, Ma Jie, Martin Glen P, McLernon David J, Andaur Navarro Constanza L, Reitsma Johannes B, Sergeant Jamie C, Shi Chunhu, Skoetz Nicole, Smits Luc J M, Snell Kym I E, Sperrin Matthew, Spijker René, Steyerberg Ewout W, Takada Toshihiko, Tzoulaki Ioanna, van Kuijk Sander M J, van Bussel Bas C T, van der Horst Iwan C C, van Royen Florien S, Verbakel Jan Y, Wallisch Christine, Wilkinson Jack, Wolff Robert, Hooft Lotty, Moons Karel G M, van Smeden Maarten Prediction models for diagnosis and prognosis of covid‐19: systematic review and critical appraisal. BMJm1328(2020). 10.1136/bmj.m1328 32265220 PMC7222643

[19] Van Calster Ben, Vickers Andrew J. Calibration of Risk Prediction Models. Medical Decision Making 35, (2), 162–169 (2015). 10.1177/0272989x14547233 25155798

[20] Enfield, Kyle, Miller, Russ, Rice, Todd, Thompson, B Taylor:Truwit, Jonathon, et al.: Limited utility of SOFA and APACHE II prediction models for ICU triage in pandemic influenza. Chest 140(4), 913A (2011). 10.1378/chest.1118087

[21] NHS England & NHS Improvement. (2020). Specialty guides for patient management during the coronavirus pandemic Guidance for the role and use of non‐invasive respiratory support in adult patients with coronavirus (confirmed or suspected). Available at: https://www.nice.org.uk/guidance/ng159 (Accessed: 05 April 2020).

[22] The RECOVERY Collaborative Group: Dexamethasone in hospitalized patients with Covid‐19. New England Journal of Medicine 384(8), 693–704 (2021). 10.1056/NEJMoa2021436 PMC7383595 32678530

[23] O'Reilly Nugent Andrew, Kelly Paul T., Stanton Josh, Swanney Maureen P., Graham Bruce, Beckert Lutz Measurement of oxygen concentration delivered via nasal cannulae by tracheal sampling. Respirology 19, (4), 538–543 (2014). 10.1111/resp.12268 24661379

[24] Wagstaff T. A. J., Soni N. Performance of six types of oxygen delivery devices at varying respiratory rates*. Anaesthesia 62, (5), 492–503 (2007). 10.1111/j.1365-2044.2007.05026.x 17448063

[25] Malycha James, Farajidavar Nazli, Pimentel Marco A.F., Redfern Oliver, Clifton David A., Tarassenko Lionel, Meredith Paul, Prytherch David, Ludbrook Guy, Young J.Duncan, Watkinson Peter J. The effect of fractional inspired oxygen concentration on early warning score performance: A database analysis. Resuscitation 139, 192–199 (2019). 10.1016/j.resuscitation.2019.04.002 31005587 PMC6547016

[26] Shamout Farah E., Zhu Tingting, Sharma Pulkit, Watkinson Peter J., Clifton David A. Deep Interpretable Early Warning System for the Detection of Clinical Deterioration. IEEE Journal of Biomedical and Health Informatics 24, (2), 437–446 (2020). 10.1109/jbhi.2019.2937803 31545746

[27] Chang, D., et al.: Risk prediction of critical vital signs for ICU patients using recurrent neural network. In: 2019 International Conference on Computational Science and Computational Intelligence (CSCI), IEEE, pp. 1003–1006 (2019)

[28] Tonekaboni, S., et al.: What clinicians want: Contextualizing explainable machine learning for clinical end use. arXiv:190505134 (2019)

