# Supplementary material for: Development and validation of early warning score systems for COVID‐19 patients
Source: Healthc Technol Lett. 2021 May 27;8(5):105–17. doi: 10.1049/htl2.12009 (PMC8239612; doi:10.1049/htl2.12009)
Supplement: Supplementary file 1 — Supporting Information [file HTL2-8-105-s001.pdf]

Supplementary materials

Table 1: The descriptive statistics of the study dataset

**Variable**	**Value**
Age (mean±std)	67.85±16.45
Age >45 (%)	91.11%
Female (%)	46.99%
Admissions (n)	472
Events (n)	137

Table 2: Mean and IQR of Vital Signs in the study dataset

**Vital Sign**	**Mean (IQR)**
Heart Rate (bpm)	89.7 (77.0-100.0)
Respiratory Rate (brpm)	21.4 (18.0-24.0)
Systolic Blood Pressure (mmHg)	127 (113-140)
SpO_2 (%)	93.9 (93.0-96.0)
Temperature (°C)	37.1 (36.5-37.7)

Table 3: Mean and IQR of Blood Tests in the study dataset

**Test**	**Mean (IQR)**
Alanine Aminotransferase (IU/L)	33.6 (18.0-40.0)
Albumin (g/L)	27.8 (24.0-31.0)
Alkaline Phosphatase (IU/L)	97.4 (62.0-111.5)
Basophils (x10^9/L)	0.02 (0.01-0.02)
Bilirubin (μmol/L)	10.1 (6.0-12.0)
C-reactive Protein (mg/L)	148.8 (84.9-210.8)
Creatinine (μmol/L)	148.5 (65.0-108.0)
Eosinophils (x10^9/L)	0.03 (0.00-0.03)
Haematocrit (L/L)	0.36 (0.32-0.43)
Haemoglobin (g/L)	119.4 (102.0-141.0)
Lymphocytes (x10^9/L)	1.0 (0.4-1.0)
Mean Cellular Volume (fL)	90.4 (86.7-94.6)
Monocytes (x10^9/L)	0.47 (0.22-0.57)
Neutrophils (x10^9/L)	6.21 (4.02-8.01)
Platelets (x10^9/L)	199.2 (140.0-230.5)
Potassium (mmol/L)	4.0 (3.7-4.2)
Sodium (mmol/L)	134.7 (132.0-138.0)
Urea (mmol/L)	8.9 (4.5-9.3)
White Blood Cells (x10^9/L)	7.8 (4.9-9.4)

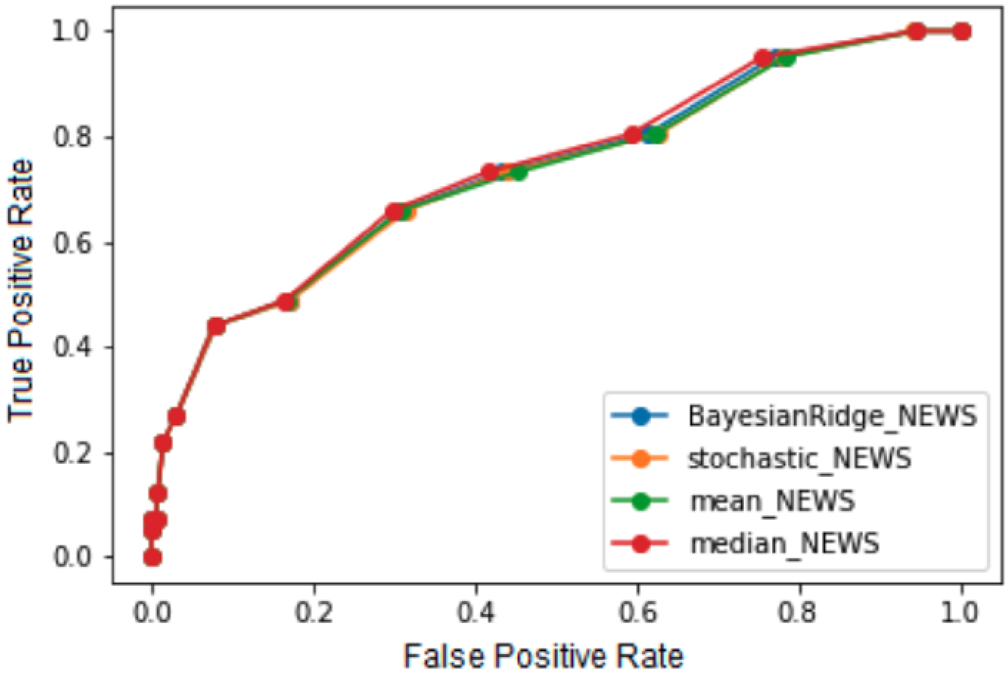

Figure 1: Performance of different imputation techniques for the NEWS score (As measured by the Area Under Receiver Operating Curve). The median technique has slightly outperformed the other imputation techniques. We have used the median as the imputation technique in our study. The x-axis is the False Positive Rate and the y-axis is the True Positive Rate

Table 4: Mean and IQR of Blood Gases in the study dataset

**Test**	**Mean (IQR)**
BE ACT	0.99 (-0.80-3.3)
BE STD	1.05 (-1.10-3.60)
Bicarbonate	24.9 (22.8-26.7)
Calcium	1.10 (1.06-1.15)
cLac	1.6 (1.0-1.8)
Creatinine	170.1 (77.2-128.0)
CtO_2C	12.3 (8.0-16.7)
Est. Osmolality	278.9 (271.6-284.6)
Fraction COHb	0.95 (0.70-1.10)
Fraction HHb	27.78 (6.70-47.70)
FIO_2	24.2 (21.0-21.0)
Glucose	8.0 (5.8-8.7)
Haemoglobin	125.5 (109.0-146.0)
Haematocrit	38.5 (33.7-44.7)
Potassium	4.0 (3.7-4.2)
Met. Hemoglobin	0.7 (0.4-0.8)
Sodium	135.2 (132.0-138.0)
O_2 Saturation	71.0 (49.4-93.2)
P5OC	3.6 (3.3-3.9)
pCO_2	5.15 (4.52-5.72)
pH	7.43 (7.39-7.47)
pO_2	7.37 (3.94-8.74)
Temperature	37.1 (37.0-37.0)

Table 5: Different levels of O2 support and the corresponding oxygen support devices in each category.

Level of O2 Support	Device Name
Level 0	Room air (No Device support)
Level 1	Nasal Cannulae, Simple Mask, Venturi Face Mask, Nebuliser Mask, Oxy-mask, Tracheostomy Mask
Level 2	Non-rebreather mask, Reservoir Mask
Level 3	High flow, Non-invasive system, CPAP, Airvo

Table 6: Classification methods, their parameter settings, and pros and cons.

Method	Summary	Parameter setting	Pros and cons
LR	LR is a linear model that optimises a set of weights for each feature that lead to the best classification performance.	This model was performed using LIBLINEAR library.	LR is easy to implement and efficient to train. However LR cannot solve non-linear problems and has high bias.
RF	RF is an averaging method that is based on building several independent Decision Tree (DT) classifiers on different subsets of the dataset and average results to produce final predictions and improve the performance.	100 estimators were considered for RF training.	It is based on training of several independent trees that can be fit in parallel. Moreover, RF usually reduce the variance. However, it needs heavier computational resources.
GBT	GBT is a boosting method based on sequentially building weak estimators (models that are only slightly better than the random prediction, i.e. small DTs). Initially, all data samples have the same weight. However, at successive iterations, the weights of mislabelled training samples by the boosted model at the previous step are increased, while the weights are decreased for the remaining training samples. Hence, as iterations proceed, the influence of difficult samples is increased. Later, it combines the predictions through a weighted majority vote with the aim of reducing the bias of the combined classifier.	DT is used as the base classifier and Binomial deviance and 100 estimators were considered for the training.	It is more robust to outliers and has high predictive power. Nonetheless, due to the sequential nature, it cannot be parallelised.
